# Epidemiological characteristics, management, and outcomes of atrial fibrillation in TUNISIA: Results from the National Tunisian Registry of Atrial Fibrillation (NATURE‐AF)

**DOI:** 10.1002/clc.23558

**Published:** 2021-03-11

**Authors:** Sana Ouali, Afef Ben Halima, Sonia Chabrak, Rafik Chettaoui, Manel Ben Halima, Abdeddayem Haggui, Salma Krichane, Larbi Noureddine, Sonia Marrakchi, Selma Charfeddine, Majed Hassine, Khaled Sayahi, Fehmi Abbes Mohamed, Wided Nasraoui, Hassen Ajmi, Mehdi Ben Miled, Zeynab Jebbari, Mohamed Ali Meghaieth, Emna Allouche, Rachid Mechmeche, Lilia Zakhama, Wissem Sdiri, Ali Ben Khalfallah, Anissa Gharbi, Sami Milouchi, Ali Neji, Saoussen Antit, Kais Battikh, Meriem Drissa, Samira Kaabachi, Tarek Najar, Rami Tlili, Iheb Chahbani, Hanene Charfeddine, Mbarek Mohamed Ben, Sami Braham, Faouzi Maatouk, Salem Abdesselem, Mokdad Ayari, Riadh Garbaa, Nabil Hamrouni, Dorra Mbarek, Hajer Rekik, Hamda Zaghdoudi, Wacef Ayadi, Feriel Baraket, Karim Ben Brahim, Mariem Ben Romdhane, Habib Bousadia, Wassim Brahim, Malek Mezri, Ali Guesmi, Taha Ounissi, Sofiene Kammoun, Wajih Smati, Samir Tlili, Karim Zoughi, Jawher Zemni, Mahmoud Cheikh Bouhlel, Sanaa Islem, Rym Jemli, Anissa Joulak, Khadija Mzoughi, Hela Naanea, Leila Hached, Moufid Hadrich, Mohamed Hmem, Slim Kacem, Ikram Kammoun, Rawdha Othmani, Amel Ouerghi, Syrine Abid, Ridha Ennouri, Sandrine Haidar, Sihem Heraiech, Monia Jammali, Mourad Jarrar, Leila Riahi, Basma Trimech, Med Ali Azaiez, Foued Azzouzi, Khaled Ben Jemaa, Oussema Ben Rejab, Rim Chrigui, Wejdene Wechtati, Essia Boughzela, Gouider Jridi, Leila Bezdah, Sondes Kraiem, Habiba Drissa, Soraya Ben Youssef, Wafa Fehri, Salem Kachboura, Habib Gamra, Samir Kammoun, Mohamed Sami Mourali, Faouzi Addad, Leila Abid

**Affiliations:** ^1^ Hôpital La Rabta Tunis Tunisia; ^2^ Abderrahman Mami Pneumology and Phthisiology Hospital Ariana Tunisia; ^3^ Pasteur Clinic Tunis Tunisia; ^4^ Manar Clinic Tunis Tunisia; ^5^ Hôpital Militaire Principal d'instruction de Tunis Tunis Tunisia; ^6^ El Alya Clinic Sfax Tunisia; ^7^ Hedi Chaker Hospital Sfax Tunisia; ^8^ Fattouma Bourguiba University Hospital Monastir Tunisia; ^9^ Mhamed Bourguiba Hospital El Kef Tunisia; ^10^ Zaghouan Hospital Zaghouan Tunisia; ^11^ Kasserine Hospital Kasserine Tunisia; ^12^ Habib Bourguiba Hospital Medenine Tunisia; ^13^ Zaghouan Clinic Zaghouan Tunisia; ^14^ Djerba Clinic DJerba Tunisia; ^15^ Charles Nicolle Hospital Tunis Tunisia; ^16^ La Marsa Internal Security Forces Hospital La Marsa Tunisia; ^17^ Bougatfa Hospital Bizerte Tunisia; ^18^ Menzel Bourguiba Hospital Bizerte Tunisia; ^19^ Essalem Clinic Sousse Tunisia; ^20^ Hopital Habib Bourguiba Medenine Tunisia; ^21^ Ben Guerdane Hospital Medenine Tunisia; ^22^ Djerba Clinic Medenine Tunisia; ^23^ Cap Bon Clinic Nabeul Tunisia; ^24^ University Hospital Center Mongi Slim La Marsa Tunisia; ^25^ Kebili Hospital Kebili Tunisia; ^26^ El Amen Clinic Beja Tunisia; ^27^ Cardiovascular Clinic Tunis Tunisia; ^28^ Carthagène Clinic Tunis Tunisia; ^29^ Ezzayatin Clinic Sousse Tunisia; ^30^ Mohamed Ben Sassi Hospital Gabes Tunisia; ^31^ Mohamed Taher Al Maamouri Hospital Nabeul Tunisia; ^32^ Habib Thameur Hospital Tunis Tunisia; ^33^ Hopital Regional de Gafsa Gafsa Tunisia; ^34^ Hédi Jaballah Hospital Tozeur Tunisia; ^35^ Essalem Clinic Tunis Tunisia; ^36^ The Berges of Lac Clinic Tunis Tunisia; ^37^ Carthage Clinic Monastir Tunisia; ^38^ El Alya Polyclinic Sfax Tunisia; ^39^ Farhat Hached University Hospital Sousse Tunisia

**Keywords:** anticoagulation, atrial fibrillation, management, North Africa, outcomes, risk scores

## Abstract

**Background:**

Contemporary registries on atrial fibrillation (AF) are scare in North African countries.

**Hypothesis:**

In the context of the epidemiological transition, prevalence of valvular AF in Tunisia has decreased and the quality of management is still suboptimal.

**Methods:**

NATURE‐AF is a prospective Tunisian registry, involving consecutive patients with AF from March 1, 2017 to May 31, 2017, with a one‐year follow‐up period. All the patients with an Electrocardiogram‐documented AF, confirmed in the year prior to enrolment were eligible. The epidemiological characteristics and outcomes were described.

**Results:**

A total of 915 patients were included in this study, with a mean age of 64.3 ± 22 years and a male/female sex ratio of 0.93. Valvular AF was identified in 22.4% of the patients. The mean CHA_2_DS_2_VASC score in nonvalvular AF was 2.4 ± 1.6. Monotherapy with antiplatelet agents was prescribed for 13.8% of the patients. However, 21.7% of the subjects did not receive any antithrombotic agent. Oral anticoagulants were prescribed for half of the patients with a low embolic risk score. In 341 patients, the mean time in therapeutic range was 48.87 ± 28.69%. Amiodarone was the most common antiarrhythmic agent used (52.6%). During a 12‐month follow‐up period, 15 patients (1.64%) had thromboembolism, 53 patients (5.8%) had major hemorrhage, and 52 patients (5.7%) died.

**Conclusions:**

NATURE‐AF has provided systematic collection of contemporary data regarding the epidemiological and clinical characteristics as well as the management of AF by cardiologists in Tunisia. Valvular AF is still prevalent and the quality of anticoagulation was suboptimal.

## 1 INTRODUCTION

In the last decades, a significant change in the epidemiologic and etiologic patterns of cardiovascular diseases has been noted in North African countries, with a decrease in rheumatic heart disease and an increase in hypertensive and ischemic heart diseases.^1^, ^2^ A decrease in the incidence of acute rheumatic fever and rheumatic heart disease has also been observed in the last four decades in African countries.^3^, ^4^ It is also estimated that by 2050, prevalence of atrial fibrillation (AF) in Africa will be greater than in any other region in the world.^1^

As for all heart diseases, there are insufficient contemporary population‐based data, describing the epidemiological and management pattern of AF patients receiving routine medical care in North Africa, especially in Tunisia where rheumatic valvular disease was the most underlying etiology of AF in 2003.^5^

Demographic and prognostic AF data from other ethnic groups, such as European, Asian, and American countries would not be extrapolated to our population. It is unknown whether occidental studies could be easily applied to low‐to‐middle income regions, such as North African populations, where reported data are still scarce.

Rheumatic heart disease is present in more than fifth of African AF patients^6^, ^7^, ^8^ compared with 2% in North American AF patients.^9^ Anticoagulation prescription rates are low in African AF patients and they have decreased progressively over time. Only 33% of the patients with valvular AF and 12% of those with nonvalvular AF are on anticoagulants at the six‐month follow‐up.^8^

Thus, a register or a survey dealing with the demographic and prognostic characteristics of AF in Tunisia is essential to be able to identify its specific characteristics inherent partly to the ethnic particularities, but especially to the particularities of the local health system, in the context of the epidemiological and guidelines transitions. Hence, the National Tunisian Registry of Atrial Fibrillation (NATURE‐AF) was performed as previously described.^10^ The aim of the present registry was to describe the epidemiological characteristics, the quality of management and the outcomes over a 12‐month follow up period.

## 2 METHODS

NATURE‐AF is a prospective, observational registry with a 1‐year follow‐up period. It included consecutive in‐ and outpatients with AF presenting to cardiologists between March 1, 2017, and May 31, 2017 all over Tunisia. Screening for eligibility was performed at the time of the patients' presentation to a cardiologist (hospital or medical center). A written informed consent was obtained from all the participants. Patients aged 20 years and older and having at least 1 episode of AF recorded on a standard 12‐lead electrocardiogram or on 24‐h Holter monitoring were officially involved in NATURE‐AF. The qualifying episode of AF should have occurred within the previous year, whether it was valvular or nonvalvular AF. Valvular etiology is considered in patients with at least moderate mitral stenosis or prosthetic heart valves. Patients did not need to have AF at the time of enrollment.

The exclusion criteria were AF due to reversible causes (e.g., thyroid disease and pulmonary embolism), including postoperative AF (≤3 months), life expectancy less than 12 months, acute coronary syndrome, isolated atrial flutter, mental disorders, and ongoing anticoagulation for reasons other than AF.

The plan was to have one baseline visit and one visit every 3 months over a 1‐year period. Enrollment into the registry started on March 1, 2017, with an estimated inclusion period extending up to 3 months. All the patients were followed for 12 months. During this period, all the participants revisited their cardiologists at the usual intervals (3 months), and the patients taking oral anticoagulant therapy consulted (or visited) at least once every month to have their INR measured.

The data collected were analyzed using the Clinical Suite platform (Dacima Software), complying with the international standards, including US Food and Drug Administration 21 Code of Federal Regulations Part 11, US Health Insurance Portability and Accountability Act, International Conference on Harmonization, and Medical Dictionary for Regulatory Activities. The Clinical Suite platform allowed to track the data entered, to check for inconsistencies and missing data, and to schedule the monitoring visits. A steering committee was set up to monitor patients' inclusions, to verify data sources, to perform the audit trail, and to prepare the statistical analysis plan for the study. The steering committee was composed of the arrhythmia working‐group of the National Society of Cardiology and Cardiovascular Surgery (www.stcccv.org.tn).

Data were collected every 3 months regardless of the patients' clinical follow‐up. All incident events and therapeutic changes were entered at each collection interval.

Baseline data included the patients' demographics, medical history, cardiovascular history, details of AF history and therapies, vital signs, laboratory measurements, electrocardiographic data, cardiac imaging parameters, details of medical management, and any contraindications to anticoagulation. CHA_2_DS_2_VASc score (Congestive heart failure history, Hypertension history, Age, Diabetes history, Stroke—thromboembolism history, vascular disease history), HAS‐BLED score (Hypertension, Renal disease, Liver disease, stroke history, Prior major bleeding or disposition to bleeding, age > 65, medication usage predisposing to bleeding, and alcohol use), and SAMe‐TT2R2 score (Sex, Age, Medical history, Treatment, Tabacco use, Race) were calculated. During the follow‐up period, major incidents, events, and procedures were recorded. The INR value was also noted for patients taking acenocoumarol.

The endpoints of NATURE‐AF are to describe:

1. the epidemiological characteristics of patients with AF in Tunisia
2. the management features regarding the anticoagulation characteristics and the choice of the rate or rhythm control strategy
3. the outcomes over a 12‐month follow‐up period: incidence of thromboembolic events, cardiovascular death, and hemorrhagic accidents

### 2.1 Statistical analysis

The normality of continuous variables was verified with the Shapiro–Wilk test. Continuous variables were described by mean and standard deviation or as median and interquartile range. Categorical variables were described by the size and frequency of every modality. Means comparison was performed by analysis of variance or by nonparametric tests if the hypothesis of normality is rejected. Statistical tests were bilateral with a 95% confidence interval.

A chi‐square test was performed for categorical variables. The Yates correction or Fisher exact test was used if the conditions of validity for the chi‐square test were not met.

A multivariate analysis was performed with anticoagulant treatment (over or undertreated) as dependent factor. The independent variables were age, gender, body mass index, type of AF, and combined therapy. Univariate logistic regression was carried out with a 10% output threshold. The final model was performed using the parameters selected by the backward stepwise method of Wald. The selected variables in the final model were tested at the 5% threshold. Interaction between the selected parameters was tested at the 10% threshold.

The TTR was calculated as described by Rosendaal et al,^11^ using linear interpolation of INR values in each patient under oral anticoagulant treatment to calculate the percentage of days when INR is in the therapeutic range (2.0–3.0) for nonvalvular AF.

## 3 RESULTS

### 3.1 Patient characteristics

A total of 915 patients, including 475 (51.9%) women, were involved in NATURE‐AF. The mean (SD) age of the patients was 64.27 (22) years. Among the patients, 202 (22.1%) were aged 75 years and older, and 67.8% of them (*n* = 620) were enrolled by public cardiologists. The number of healthcare units involved was 37. A total of eighty‐eight cardiologists participated in the study, representing 22% of the total cardiologists in Tunisia. Half of the investigators were in the private sector.

The baseline clinical characteristics of all the registered AF patients are summarized in Table 1.

**TABLE 1:** Clinical characteristics and antithrombotic and antiarrhythmic drugs used of the study population

Clinical characteristics	Total (*N* = 915)	Nonvalvular AF *N* = 710. (77.6%)	Valvular AF *N* = 205 (22.4%)	*p*
Age (years) (mean ± SD)	64.27 ± 22	64.22 ± 22	64.43 ± 24	.846
Sex ratio (male/female)	0.93	1.01	0.69	.022
BMI kg/m^2^ (mean ± SD)	27.92 ± 5.06	27.94 ± 5.51	27.85 ± 17.1	.837
Smoking habit (*N* [%])	159 (17.4%)	139 (19.6%)	20 (9.8%)	.001
Dyslipidemia (*N* [%])	180 (19.7%)	144 (20.3%)	36 (17.6%)	.426
Hypertension (*N* [%])	439 (48.0%)	355 (50.0%)	84 (41.0%)	.026
Diabetes mellitus (*N* [%])	207 (22.6%)	165 (23.2%)	42 (20.5%)	.449
Congestive heart failure (*N* [%])	116 (12.7%)	91 (12.8%)	25 (12.2%)	.905
Prior stroke/TIA /thromboembolic event (*N* [%])	78 (8.5%)	57 (8.0%)	21 (10.2%)	.319
Age ≥ 75 years (*N* [%])	202 (22.1%)	152 (21.4%)	50 (24.4%)	.365
Age ≥ 65 years (*N* [%])	460 (50.3%)	356 (50.1%)	104 (50.7)	.937
LVEF (%) (mean ± SD)	58.1 ± 12.3	58.2 ± 12.6	57.4 ± 11.6	.516
Coronary artery disease (*N* (%))	95 (10.4%)	77 (10.8%)	18 (8.8%)	.393
Prior PCI (*N* (%))	55 (6%)	47 (6.6%)	8 (3.9%)	.149
Prior CABG (*N* (%))	16 (1.7%)	12 (1.7%)	4 (2.0%)	.775
Clearance <30 ml (*N* (%))	13 (1.5%)	9 (1.2%)	4 (2.0%)	.657
Cancer (*N* (%))	18 (2.0%)	14(2.0%)	4(2.0%)	.977
History of stroke (*N* (%))	78 (8.5%)	57 (8.0%)	21 (10.2%)	.319
History of bleeding (*N* (%))	17 (1.9%)	15 (2.1%)	2 (1.0%)	.306
Obstructive sleep apnea (*N* (%))	26 (2.8%)	19 (2.7%)	7 (3.4%)	.575
Respiratory failure (*N* (%))	49 (5.5%)	39 (5.5%)	10 (4.9%)	.731
Paroxysmal AF (*N* (%))	308 (33.7%)	268 (37.7%)	40 (19.5%)	<.001
Persistent AF (*N* (%))	313 (34.2%)	237 (33.4%)	76 (37.1%)	<.001
Permanent AF (*N* (%))	206 (22.5%)	140 (19.7%)	66 (32.2%)	<.001
Palpitations (*N* (%))	529 (57.8%)	417 (58.7%)	112 (54.6%)	.296
Dyspnea (*N* (%))	358 (39.1%)	280 (39.4%)	78 (38.0%)	.720
Syncope (*N* (%))	38 (4.2%)	28 (3.9%)	10 (4.9%)	.555
CHA_2_DS_2_VASc score (mean ± SD)		2.4 ± 1.6		
Points 0		87 (12.3%)		
Points 1		143 (20.1%)		
Points ≥ 2 (%)		490 (67.6%)		
HASBLED score ≥ à 3		108 (15.2%)		
Pacemaker/ICD (*N* (%))	36 (3.9%) /6 (0.7%)	31 (4.4%) /5 (0.7%	5 (2.4%)/1 (0.5%)	.212/.999
History of Electrical cardioversion	9 (1.0%)	4 (0.6%)	5 (2.4%)	.061
Antiplatelet drugs (*n* (%))	126 (13.8%)	101 (14.2%)	25 (12.2%)	.457
Anticoagulant drug (*n* (%))	644 (70.4%)	492 (69.3%)	152 (74.1%)	.180
No antithrombotic drugs (*n* (%))	199 (21.7%)	163 (23.0%)	36 (17.6%)	.098
Combination therapy of antiplatelet agents and oral anticoagulation, (*n* (%))	54 (5.9%)	46 (6.5%)	8 (3.9%)	.168
Beta‐blockers (*n* (%))	396 (43.9%)	302 (42.5%)	94 (45.9%)	.398
Digoxin (*n* (%))	131 (14.3%)	98 (13.8%)	33 (16.1%)	.409
Calcium inhibitors (*n* (%))	154 (16.8)	119 (16.8%)	35 (17.1%)	.916
Electrical cardioversion (*n* (%))	3 (0.3%)	0	3 (1.5%)	.022
Amiodarone, (*n* (%))	481 (52.6%)	387 (54.5%)	94 (45.9%)	.029
Flecainide, (*n* (%))	80 (8.7%)	65 (9.2%)	15 (7.3%)	.412
d,I‐Sotalol, (*n* (%))	30 (3.3%)	25 (3.5%)	5 (2.4%)	0.443
TTR % (mean) In 341 patients (37.3%)	48.87 ± 28.69	43.97 ± 27.7 206 patients (60.4%)	56.36 ± 28.55 135 patients (39.6%)	<.001
TTR ≥65% (*n* (%))	110/341 (32.3%)	47/ 206 (22.8%)	63/135 (46.7%)	<.001
SAMe‐TT2R2 score (0–2)		203 (28.6%)		

Abbreviations: AF: atrial fibrillation, BMI: body mass index, CABG: coronary artery bypass graft, CHA_2_DS_2_VASc score: [Congestive heart failure history, Hypertension history, Age, Diabetes history, Stroke – thromboembolism history, vascular disease history], EHRA: The European Heart Rhythm Association, HASBLED score: [Hypertension, Renal disease, Liver disease, stroke history, Prior major bleeding or disposition to bleeding, age > 65, medication usage predisposing to bleeding, alcohol use], ICD: implantable cardioverter defibrillator, LVEF: left ventricle ejection fraction, PCI: percutaneous coronary intervention, SAMe‐TT2R2 score: [Sex, Age, Medical history, Treatment, Tabacco use, Race], TTR: time in therapeutic range.

Among the included patients, 33.7% of them were in paroxysmal AF, 34.2% were in persistent AF, and 22.5% were in permanent AF. Two hundred five patients (22.4%) had valvular atrial fibrillation. No significant age differences were noted in the AF subtypes. Fifty nine percent (59.0%) of the patients were women in the valvular AF group. The paroxysmal type of AF was significantly more frequent in the nonvalvular AF group (*p* < .001). The European Heart Rhythm Association (EHRA) score ≥ 2 was identified in 41.6% of the patients. Palpitation and dyspnea were the most frequent symptoms expressed respectively in 57.8% and 39.1% of the cohort.

The most common associated comorbidities in nonvalvular AF were hypertension (48%), obesity or overweight (53.4%), diabetes (22.6%), and congestive heart failure (12.7%).

In nonvalvular AF, the mean CHA_2_DS_2_VASc score was 2.4 ± 1.6 with 12.3% having CHA_2_DS_2_VASc score 0, 20.1% having CHA_2_DS_2_VASc score 1, and 67.6% having CHA_2_DS_2_VASc score ≥ 2 (Table 1). The mean HAS‐BLED score was 1.4 ± 1.1.

Table 1 summarizes the main characteristics of the population.

### 3.2 Use of antithrombotic agents and quality of anticoagulation (TTR)

**FIGURE 1:**
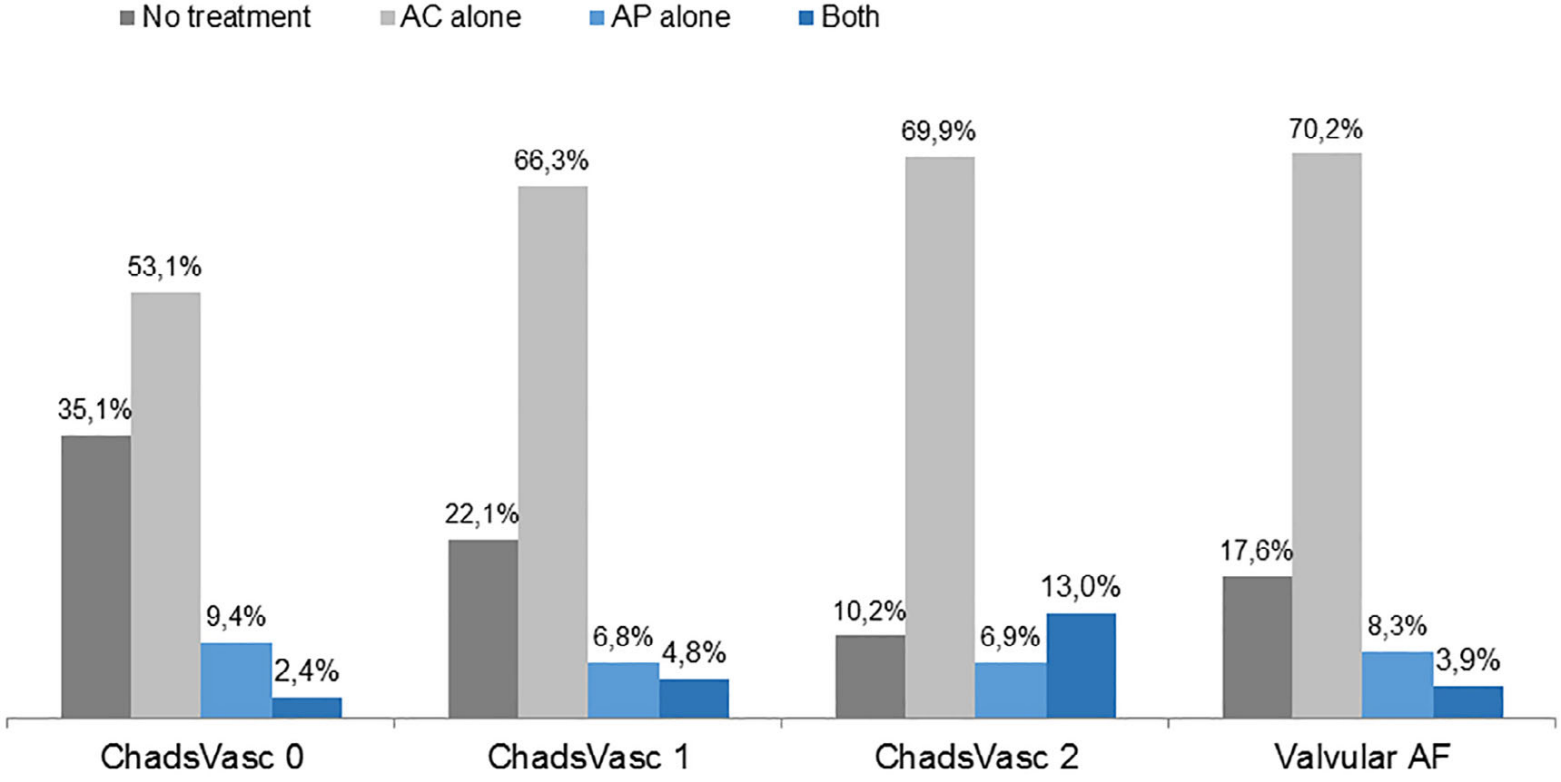
Distribution of Antithrombotic drug use in valvular and nonvalvular AF patients according to CHA_2_DS_2_VASc score

Six hundred forty four patients (70.38%) were taking anticoagulants, predominantly acenocoumarol. New oral anticoagulant drugs (NOAC) were used in only three patients. Antiplatelet agents alone were given to 126 patients (13.8%) and in combination with anticoagulants to 54 patients, accounting for 5.9% of the cohort. Among the patients, 199 (21.7%) had no antithrombotic therapy. Fifty‐three out of 205 patients with valvular AF (25.85%) had no antithrombotic therapy (36 patients) or received antiplatelet drugs (17 patients) (Figure 1).

In the group of nonvalvular AF, for patients with CHA_2_DS_2_VASc score zero (245 patients), oral anticoagulants were used in 136 patients (55.5%), antiplatelet therapy alone was prescribed in 23 patients (9.4%), and 86 patients (35.1%) did not have any antithrombotic therapy (Figure 1). In patients with *score CHADS VASC ≥ 2* (216 patients), 82.9% (179 patients) of them received anticoagulants. Seven percent (15 patients) received antiplatelet therapy alone and 10.2% (22 patients) did not have any antithrombotic therapy.

The proportions of antithrombotic use according to CHA_2_DS_2_VASc score in nonvalvular AF are shown in Figure 1.

The mean TTR with the Rosendaal method, obtained in 341 patients was 48.87 ± 28.69%. Only 110 patients (32.25%) had an adequate level of anticoagulation (TTR ≥65%).

None of the cardiovascular risk factors (hypertension, diabetes mellitus, dyslipidemia, and smoking) was significantly associated with TTR (Table 2). For patients with nonvalvular AF, none of CHA_2_DS_2_VASc score, HAS‐BLED score, or SAMe‐TT2R2 score was significantly associated with the quality of anticoagulation (TTR≥65%).

**TABLE 2:** Predictive factors for obtaining a correct time in therapeutic range for patients under coumarins (multivariable analysis)

	OR (95% CI)	*p*
Age	1.011 (0.981; 1.042)	.476
Dyslipidemia	1.345 (0.651; 2.777)	.424
Diabetes	1.484 (0.555; 3.965)	.432
Smoking	0,612 (0.22; 1.706)	.348
**CHF**	0.239 (0.065; 0.876)	**.031**
OSA	1.187 (0.209; 6.73)	.847
CHA_2_DS_2_VASc risk score	0.925 (0.471; 1.819)	.822
Hypertension	0.895 (0.348; 2.299)	.818
LVEF	0.996 (0.969; 1.023)	.745
**AP**	3.599 (1.249; 10.373)	**.018**
Cancer	0.343 (0.041; 2.848)	.322
BMI	0.972 (0.919; 1.027)	.306
Male gender	0.965 (0.469; 1.984)	.923
History of ischemic stroke	1.322 (0.507; 3.448)	.568
**Nonvalvular AF**	0.241 (0.134; 0.435)	**<.001**
SAMe‐TT2R2 score	1.2 (0.464; 3.106)	.707

Abbreviations: AP: antiplatelet, BMI: body mass index, CHF: congestive heart failure, 95% CI: 95% Confidence Interval, LVEF: left ventricular ejection fraction, OR: Odds‐ratio, OSA: obstructive sleep apnea.

After multivariate adjustment (Table 2), the variables significantly associated with poor anticoagulation level were congestive heart failure (Adjusted OR equal to 0.23, 95% CI: 0.065–0.876), and nonvalvular AF type (Adjusted OR: 0.24, 95% CI: 0.134–0.435).

However, prescription of antiplatelet therapy was associated with an adequate anticoagulation therapy (TTR≥65%) (Adjusted OR equal to 3.599, 95% CI: 1.249–10.373).

During follow‐up, 31 patients stopped anticoagulant drugs. The reasons for discontinuation were related to the patients' nonadherence in 32.3% of the cases and to the physicians' decision in 67.8% of the cases (physician preference in 45.2% of the cases and contraindication to anticoagulant in 22.6% of the patients).

### 3.3 Rate and rhythm control

Rate control was attempted in 48.4% of AF patients with the use of beta‐blockers, digoxin, and calcium blockers in respectively 59.9%, 29.9%, and 20.3% of the patients.

The most often prescribed antiarrhythmic drugs (AADs) were amiodarone (52.6%), flecainide (8.7%), and sotalol (3.3%). Catheter ablation was only attempted in 0.4% of the overall population. Pacemaker implantation was performed in 3.9% of the whole cohort.

### 3.4 Outcomes at 12 months

During the 12‐month follow‐up period, 15 patients showed thromboembolic complications (1.64%), among them two patients were not receiving anticoagulation therapy. Stroke represented 53.3% of these complications. After multivariate adjustment (Table 3), the variables significantly associated with thromboembolic complications were obstructive sleep apnea (Adjusted OR equal to 42.486, 95% CI: 4.21–428.781, *p* = .001), and higher CHA_2_DS_2_VASc score (Adjusted OR equal to 4.631, 95% CI: 1.135–18.886, *p* = .033).

**TABLE 3:** Predictive factors for thromboembolic and bleeding complications and cardiovascular death (Multivariable analysis)

	Thromboembolic complications adjusted OR (95% CI)	Bleeding complications adjusted OR (95% CI)	Cardiovascular death adjusted OR (95% CI)
Age	0.985 (0.923; 1.052)	0.991 (0.952; 1.031)	0.997 (0.955; 1.04)
Dyslipidemia	0.232 (0.015; 3.595)	1.12 (0.409; 3.062)	0.949 (0.32; 2.814)
Diabetes mellitus	0.1 (0.009; 1.141)	0.936 (0.218; 4.016)	0.457 (0.125; 1.674)
Smoking habit	0.861 (0.084; 8.849)	0.894 (0.309; 2.59)	1.224 (0.406; 3.692)
Congestive heart failure	2.348 (0.377; 14.624)	0.496 (0.103; 2.38)	**3.821 (1.377; 10.607)#**
Obstructive sleep apnea	42.486 (4.21; 428.781)*	1.619 (0.254; 10.319)	
Mean CHA_2_DS_2_VASc score	4.631 (1.135; 18.886)**	0.626 (0.228; 1.719)	3.147 (1.32; 7.5)
Hypertension	0.435 (0.056; 3.392)	**4.027 (1.065; 15.227)§**	**0.374 (0.108; 1.291)#**
LVEF	0.998 (0.931; 1.069)	0.991 (0.953; 1.03)	1.024 (0.984; 1.066)
Oral Anticoagulant drugs	0.533 (0.078; 3.659)	2.367 (0.657; 8.527)	1.506 (0.458; 4.947)
Antiplatelet drugs	0.699 (0.085; 5.748)	**4.659 (1.509; 14.381)§§**	1.83 (0.577; 5.804)
Respiratory failure	0.807 (0.073; 8.88)	1.487 (0.35; 6.31)	0.741 (0.153; 3.579)
Cancer	4.266 (0.223; 81.65)	2.682 (0.515; 13.972)	2.954 (0.459; 19.022)
Body mass index	0.84 (0.704; 1.002)	0.965 (0.889; 1.047)	0.943 (0.862; 1.031)
Coronary artery disease	2.324 (0.211; 25.632)	0.656 (0.137; 3.147)	1.446 (0.427; 4.892)
Gender (Male)	0.715 (0.154; 3.331)	0.721 (0.304; 1.709)	0.826 (0.329; 2.071)

*Note*: *p = .001; **p = .033; §*p* = .04; §§*p* = .007; #*p* = .01. Adjusted OR: Adjusted Odds‐ratio, 95% CI: 95% Confidence Interval.

Bleeding occurred in 53 patients (5.8%). Cerebral hemorrhage and digestive bleeding were reported during the follow‐up, respectively in 0.5 and 1% of all AF patients. Hypertension (Adjusted OR equal to 4.027, 95% CI: 1.065–15.227, *p* = .04) and antiplatelet drug use (Adjusted OR equal to 4.659, 95% CI: 1.509–14.381, *p* = .007) were associated with bleeding complications (Table 3).

**FIGURE 2:**
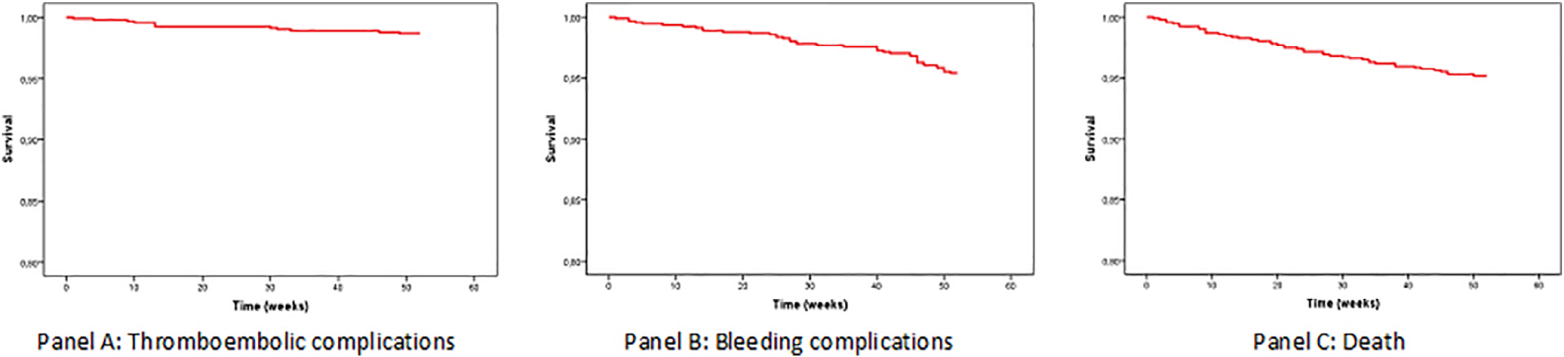
Kaplan–Meier plot: Thromboembolic complications (Panel A), Bleeding complications (Panel B) and death (Panel C) during follow‐up (survival in weeks (Moy, IC 95%) 62.4 [59.6–65.3])

During follow‐up, 52 patients died (5.6%) (Figure 2). Cardiovascular death occurred in 38 patients (4.1%). The predictive factors for cardiovascular death were congestive heart failure (Adjusted OR equal to 3.821, 95% CI: 1.377–10.607, *p* = .01), and hypertension (Adjusted OR equal to 0.374, 95% CI: 0.108–1.291, *p* = .01).

## 4 DISCUSSION

NATURE‐AF is the first large scale prospective registry assessing the epidemiological characteristics, management, and outcomes in North African patients with recently documented atrial fibrillation by both private and public cardiologists. First, valvular AF was still prevalent (22.4%) despite the demographic transition in the region. Second, the quality of anticoagulation was still poor and adherence to guidelines was not optimal. Third, Rhythm control was attempted in 48.4% of the patients and only four of them had catheter ablation. Finally, thromboembolic, bleeding, and cardiovascular death occurred in 1.64%, 5.8%, and 5.7% of the patients respectively, during the one‐year follow‐up period.

This registry provides interesting data allowing to compare treatment and response variation among AF populations in Africa and to evaluate adherence to recent guidelines.

### 4.1 Clinical characteristics

Our population was younger and characterized by more prevalence of women and valvular AF compared to non‐African AF (occidental) registries.^12^, ^13^, ^14^ In nonvalvular AF patients, the stroke risk score was lower than in ORBIT‐AF,^14^ GLORIA,^15^ GARFIELD,^16^ and PREFER^17^ registries (mean CHADSVASC score 2.4 ± 1.6).

Only two published studies describing the epidemiological data in Tunisia are available.^5^, ^7^, ^18^ In 2003, Drissa et al^5^ conducted a multicenter study involving 1134 patients presenting with a first episode of AF between January 1985 and December 2000. The average age was 58.6 (15–60) years, and 656 patients (57.8%) were males. The most common identified etiology of AF was rheumatic carditis (36.1%). Higher morbidity and mortality were demonstrated in AF patients with a 5‐year survival rate of 85%.

Thus, a decrease in the prevalence of valvular atrial fibrillation and an increase in the prevalence of nonvalvular atrial fibrillation with a high prevalence of hypertension, congestive heart failure, and diabetes were noted in NATURE‐AF. This is in accordance with the epidemiological transition seen in North Africa.

### 4.2 Antithrombotic AF management

In a recent review, Mazurek et al^19^ demonstrated that despite the differences in design and methodology between a wide variety of registries on AF in European, North American, and Asian populations, there are apparent regional differences and gaps in stroke prevention, with approximately one third of AF patients not being treated in accordance with guidelines.^20^

In our registry, 64.8% of low‐risk stroke patients received anticoagulants and/or antiplatelet therapy. In valvular AF patients and high‐risk stroke nonvalvular AF patients, 25.9% and 17.1% had no anticoagulant therapy or antiplatelet therapy, respectively.

Acenocoumarol remains the most prescribed anticoagulant (99% of the total anticoagulants prescribed in NATURE‐AF). Direct oral anticoagulants were not available in Tunisia during the inclusion and the follow‐up period of the registry. Currently, direct oral anticoagulants are gradually replacing vitamin K antagonist in both Europe and North America.^21^

Among REALIZE‐AF patients with a CHADS_2_ score ≥ 2, there are also important geographical differences with respect to the use of antithrombotic agents. In fact, the proportion of patients not receiving antithrombotic therapy ranges from 11.4% in the Middle East and Africa to 27.6% in Latin America. Conversely, the use of oral anticoagulants is the highest in the Middle East and Africa (66.7%) and the lowest in Asia (31.7%).^7^, ^18^

Despite efforts, adherence to thromboprophylaxis guidelines in NATURE‐AF was still suboptimal. This was also noticed in other registries as approximately half of truly low‐risk patients are over‐treated with oral anticoagulation, and one third of high‐risk patients are not anticoagulated.^12^, ^15^, ^22^

In NATURE‐AF, the quality of anticoagulation was still poor, with a mean TTR of 48.87 ± 28.69 and a low rate of adequate quality of anticoagulation (TTR > 65%) observed in 32.3% of the patients. The same results were obtained in a recent study published in Tunisia.^23^

In the J RHYTHM Registry, despite an overall high oral anticoagulation of 87.3%, only 53% of the patients met the target international normalized ratio levels.^24^

### 4.3 Rate versus rhythm management

Of the two main strategies for the treatment of AF, the 'rate control' and 'rhythm control' were similarly chosen in our registry. In REALIZE‐AF,^25^ the rhythm control was the most commonly chosen strategy (57.5% vs. 37.2%).

Regarding the rate control drugs, beta‐blockers and nondihydropyridine calcium channel blockers were more often used than digoxin in NATURE‐AF. Amiodarone was the most common antiarrhythmic drug used (36%). The low rate of catheter AF ablation is mainly due to economic reasons. In fact, in North African countries, costs of catheter AF ablation are borne by the patient rather than a health insurance.

In the ReLY‐AF registry,^8^ beta‐Blockers were prescribed in 41.8% of the patients, ranging from 21.7% in Africa to half of the patients in North America and Western as well as Eastern Europe. A quarter of the patients received digoxin, ranging from 12.7% in Western Europe to more than one‐third of the patients in India and Africa. In total, 8.2% of the patients were treated with verapamil or diltiazem, with only 2.0% in Africa and up to 18.5% in North America. Amiodarone was the most specific antiarrhythmic drug used in 8.7% of the patients, with a large variation from ≤5% in North America, Western Europe, the Middle East, and Africa to >5% in South America, and Eastern Europe. In EORAP‐AF pilot registry,^26^ amiodarone was the most commonly used antiarrhythmic drug (21.5%), followed by sodium channel blockers. In the EURO HEART SURVEY (EHS),^27^ like in AFNET^28^ and in PREFER^17^ registries, one third of the patients received class III antiarrhythmic agents.

### 4.4 Outcomes

AF remains a major cause of morbidity and mortality. At 1 year, cardiovascular mortality was estimated to be 5.7% and thromboembolic complications to be 1.6% in this registry. These results were similar to EORP‐AF pilot registry^26^ and EHS,^29^ where mortality was estimated to be 5.7% and 5.3%, respectively. The thromboembolic complications rate was similar to that revealed in EHS (1.8%) but higher than that shown in EOARP‐AF stroke complications.^21^, ^26^, ^29^

### 4.5 Limitations

Our data should be interpreted in the context of their limitations. In fact, this study was conducted by only 92 private and public cardiologists who accepted to participate in this registry. Only patients who consented to the study were enrolled. Therefore, not all new documented AF patients were involved, particularly those who presented to first care medical centers (noncardiologists). The follow‐up period extended over only 1 year; thus, a long term follow‐up is required.

## 5 SUMMARY

These data have important clinical and public health implications for North African populations, who are in an epidemiological transition.

Valvular AF was present in one‐fifth of Tunisian patients. Almost half of the registered nonvalvular AF patients were at low risk of stroke and had a high rate of acenoucoumarol use. However, the quality of anticoagulation was poor and compliance with the treatment guidelines remained suboptimal. Despite the low economic resources, health policy should enhance educational strategies, screening, management, and prevention strategies, in addition to new medication and technologies, such as catheter AF ablation and the use of direct oral anticoagulants to improve AF management and to offer better risk benefit ratios. These results highlight the need for a strategy that might have particular advantages for middle‐income North African countries, having limited resources.

## CONFLICT OF INTEREST

The authors declare no potential conflict of interests.

## AUTHOR CONTRIBUTIONS

The manuscript has been read and approved by all the authors.

## ETHICS STATEMENT

Ethics approval was applicable and a written informed consent was obtained from the participants in the study.

## Data Availability

Data supporting the findings of this study are available on request from the corresponding author.
